# Altitudinal dependence of PCBs and PBDEs in soil along the two sides of Mt. Sygera, southeastern Tibetan Plateau

**DOI:** 10.1038/s41598-018-32093-y

**Published:** 2018-09-19

**Authors:** Wenying Meng, Pu Wang, Ruiqiang Yang, Huizhong Sun, Julius Matsiko, Dou Wang, Peijie Zuo, Yingming Li, Qinghua Zhang, Guibin Jiang

**Affiliations:** 1 State Key Laboratory of Environmental Chemistry and Ecotoxicology, Research Center for Eco-Environmental Sciences, Chinese Academy of Sciences, Beijing, 100085 China; 2 University of Chinese Academy of Sciences, Beijing, 100049 China; 3 Institute of Environment and Health, Jianghan University, Wuhan, 430056 China

## Abstract

Surface soil samples were collected from Mt. Sygera in the southeast of Tibetan Plateau to investigate the altitudinal distribution of PCBs and PBDEs along the two sides of the mountain. The average concentrations of PCBs and PBDEs were 177 pg g^−1^ dw and 15 pg g^−1^ dw, respectively. The relationships between the log-transformed TOC-normalized concentrations and the altitudes showed different trends on the two sides. On the windward side, there was a positive correlation for the heavier PCBs; while on the leeward side, the concentrations increased and then decreased for PCBs and PBDEs at the altitude of 4100–4200 m, corresponding to the change in vegetation. The observed discrepancy on the two sides of the mountain demonstrated different key factors associated with precipitation and the forest canopy. Additionally, values of windward-leeward Enrichment Factors (W/L EFs) for the heavier PCB congeners (PCB-138, 153, and -180) were an order of magnitude higher in sites above 4200 m, which also suggested that vegetation played an important role in the altitudinal accumulation of POPs in soil. This is one of the very few studies that have revealed the differences in altitudinal accumulation of POPs along the two sides of a mountain.

## Introduction

Persistent organic pollutants (POPs) are chemical substances which are toxic, bioaccumulative, persistent in the environment, and can undergo long-range atmospheric transport (LRAT) from polluted regions to pristine areas, such as the Antarctic, Arctic and the Tibetan Plateau (TP)^1–3^. Polychlorinated biphenyls (PCBs) and polybrominated diphenyl ethers (PBDEs) are two classes of typical POPs under the Stockholm Convention, which share many similar physicochemical properties and have been found ubiquitously in the environment worldwide.

Pristine regions with temperature-driven evaporation/deposition are prone to the enrichment of POPs through global fractionation and cold condensation^4,5^. Several studies in high latitude regions, including the Arctic^6–8^ and the Antarctic^9–11^, revealed the widespread distribution of these chemicals. Similar to the “cold condensation” occurring in high latitude regions, POPs can be enriched in high-altitude areas by mountain cold-trapping. The dependence on altitude of accumulation of POPs in mountains was first reported in 1998^12^, and since then, more and more studies have been conducted on the altitudinal distribution of POPs in mountainous regions at high altitude^13–17^. Wania *et al*.^18^ put forward a specific mechanism on mountain cold-trapping, which indicated that the heavier POPs were prone to be enriched in high altitudes. However, some studies indicated that to some extent, the more volatile pollutants were enriched at higher altitude^19,20^. Tremolada^19^ found out that the distribution of heavier PCBs was negatively correlated with the altitude and both the composition and levels of POPs gave different results on two transects of a mountain. This suggested that further studies were warranted to reveal the specified accumulation of POPs in remote mountains.

**Figure 1:**
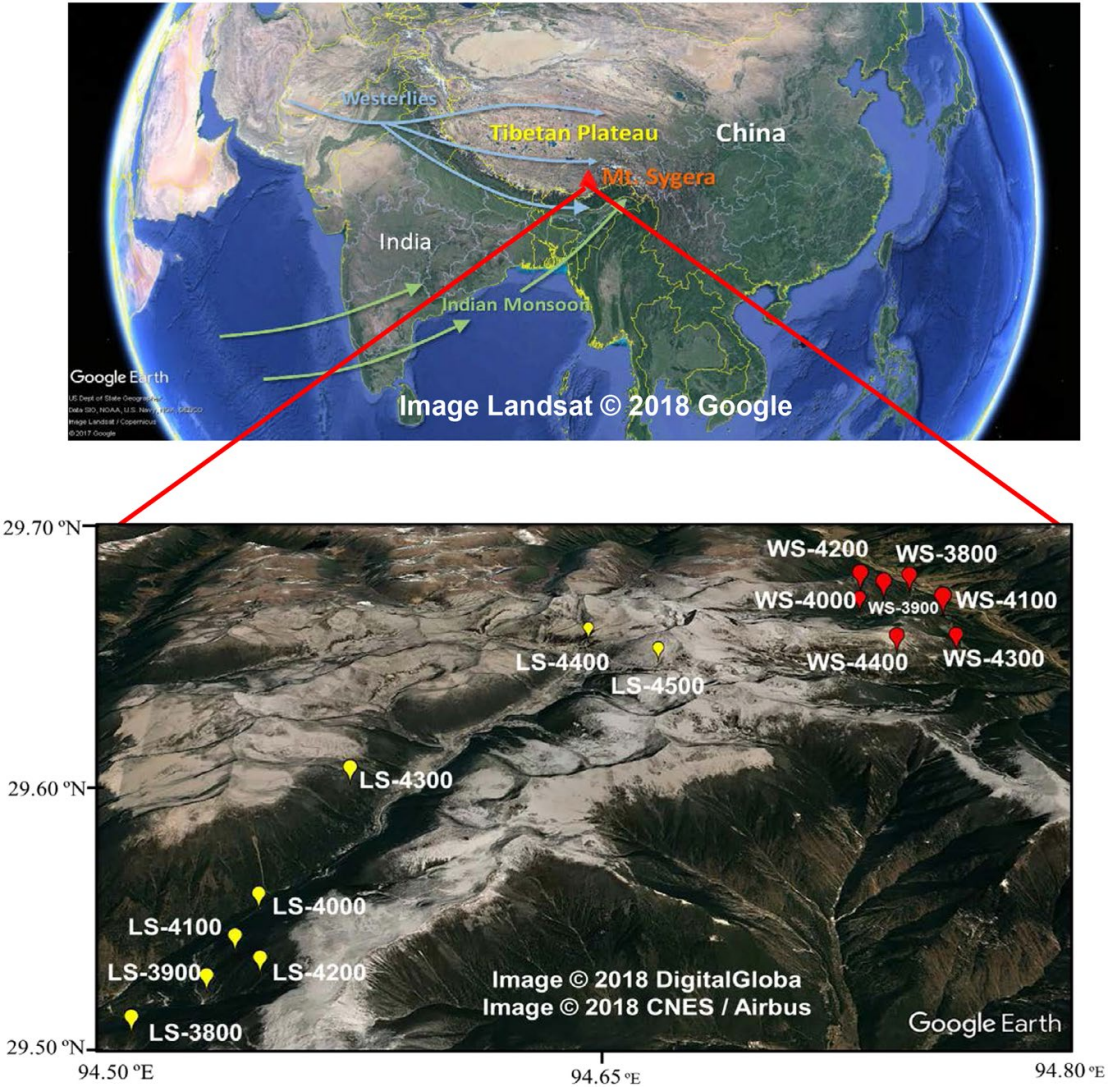
The map of the sampling sites.

The Tibetan Plateau (TP), located in the eastern Eurasian continent, is the largest and highest plateau in the world. It is regarded as the third pole because of the unique meteorological and geographic characteristics. Compared with the western and northern TP regions, the altitude of the southeastern TP is generally lower. It is characterized by mountain-valley topography and is one of the main forest regions of the plateau due to the abundant precipitation driven by Indian monsoon. This region is less impacted by human activities and POPs there are considered to have originated from distant sources^21^. Mt. Sygera lies in the southeastern TP and is characterized by the windward and leeward side, which makes it an ideal setting for studies on LRAT of POPs. Previously, Wang *et al*.^22^ investigated the distribution profiles of organochlorine pesticides (OCPs) and PCBs in the forest soil and found that there was no difference on both sides of this mountain. Zhu *et al*.^23^ investigated the altitudinal distribution of PCBs and PBDEs in air along different slopes of Mt. Sygera in 2015 and found out that the POP concentrations had no obvious variation on the windward side but observed a decreasing trend on the leeward side along the altitude. Additionally, Luo *et al*.^24^ found an increasing trend of α-HCH and DDTs with the increasing altitude on the windward side but no correlation with the altitude on the leeward side. However, the results of our previous study revealed that the concentration of OCPs increased with altitude on the windward and leeward side of Mt. Sygera, suggesting the role of forests as a filter and forest soil as a final sink^25^. In this study, we aimed to further reveal the altitudinal dependence of PCBs and PBDEs in surface soil along the two sides (windward and leeward side) of Mt. Sygera (Fig. 1). The results will strengthen the current understanding of the potential influence of a mountain on the environmental behavior and fate of POPs.

## Results

### The total organic carbon (TOC)

In this study, the TOC values were in the range of 3.60–12.36% with an average of 7.74% (Table 1). The lowest values were generally obtained at the lower altitude of the windward side (3800 m and 3900 m). However, there was no significant difference observed between the TOC values on both the sides (*p* > 0.05).

**Table 1:** Information on sampling and concentrations of PCBs and PBDEs.

No	Altitude (m a.s.l)	TOC (%)	Conc. (pg g^−1^ dw)	
			Σ_19_PCBs	Σ_16_PBDEs
WS-4400	4400	9.63	221	11.1
WS-4300	4300	12.36	268	9.3
WS-4200	4200	7.14	99	19.6
WS-4100	4100	8.23	243	31.2
WS-4000	4000	8.10	190	
WS-3900	3900	3.87	88	5.7
WS-3800	3800	3.60	131	15.4
LS-4500	4500	6.25	33	13.0
LS-4400	4400	9.60	64	7.8
LS-4300	4300	8.23	225	38.2
LS-4200	4200	9.84	177	11.4
LS-4100	4100	5.30	147	24.7
LS-4000	4000	6.84	95	9.3
LS-3900	3900	6.51	59	10.6
LS-3800	3800	9.11	80	12.8

WS, the windward side; LS, the leeward side. m.a.s.l., meter above sea level.

### The concentrations of PCBs and PBDEs

The concentrations of Σ_19_PCBs and Σ_16_PBDEs are reported based on dry weight (dw) in Table 1. For PCBs, the average concentrations of Σ_19_PCBs was 144 pg g^−1^ dw, ranging from 33 to 268 pg g^−1^ dw. This was consistent with other results from the TP^15,17,26^, but considerably lower than global background levels (5410 pg g^−1^)^27^, the values from European Alps soil (2900–13200 pg g^−1^)^28,29^ and other remote mountainous regions^30–32^. For PBDEs, BDE-17, 28 and 47 were obviously detected in all the samples, while the detection frequency of the other congeners were relatively low (<31%) (Table S1). The average concentration of Σ_19_PBDEs was 15 pg g^−1^ dw, ranging from 5.7 to 37 pg g^−1^ dw. The results were comparable to those in soils from the TP (mean 11 pg g^−1^)^17^ and the eastern TP (mean: 26 pg g^−1^)^15^, but much lower than other studies in the remote areas, e.g., the European background soils (65–12000 pg g^−1^ dw)^33^ and Russian Arctic (160–230 pg g^−1^ dw)^34^.

The average concentrations of Σ_19_PCBs were 177 pg g^−1^ dw (88 to 268 pg g^−1^ dw) and 110 pg g^−1^ dw (33–225 pg g^−1^ dw) on the windward and leeward side, respectively; while those of Σ_16_PBDEs were 16 pg g^−1^ dw (5.7 to 31 pg g^−1^ dw) and 16 pg g^−1^ dw (7.8–38 pg g^−1^ dw), respectively. The distribution of PCBs was consistent with the results obtained by Wang *et al*.^26^, but different from the investigation by Tremolada *et al*.^35^ on preferential retention of POPs on the leeward side of the Andossi Plateau.

### Homologue distribution of PCBs and PBDEs

**Figure 2:**
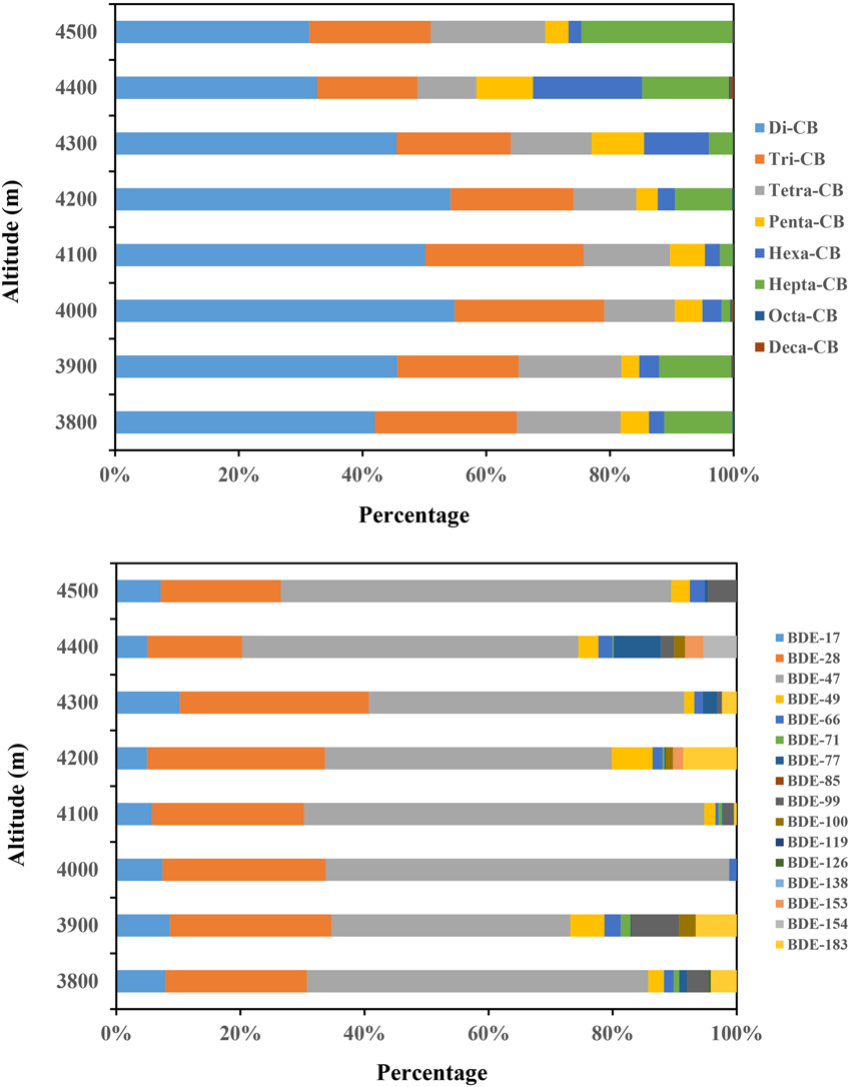
Relative contribution of PCB homologs and PBDE homologues along the altitude in Mt. Sygera.

Figure 2 displays altitudinal distribution of the PCB and PBDE homologues along the altitude of Mt. Sygera. For PCBs, seven indicator congeners were evidently detected in all samples, which accounted for more than 95% of Σ_19_PCBs. PCB-28 was the dominant congener (average 60% of the indicator PCBs). The sum of low-chlorinated congeners (di-, tri- and tetra-CB) accounted for more than 70% of ΣPCBs (Fig. 2). For PBDEs, BDE-47 was the main congener, accounting for 47% of Σ_16_PBDEs on average, followed by BDE-28 (mean 23%) (Fig. 2). This is probably because the lighter PCB and PBDE congeners are more prone to undergo LRAT^36^. Similar results have been reported in our previous works in the Balang Mountain forest, east edge of the TP^15^ and the Himalayas^17^.

## Discussion

Mt. Sygera lies in the southeastern TP. The climate in this region is subject to the southwest wind in summer (June–September) and westerly wind in winter (December–April). The valleys are usually the main channels for the warm and humid airstream from the Indian Subcontinent to the southeastern TP^37^. The temperature on both sides are similar but the windward side has more abundant vegetation due to higher precipitation and humidity^38^.

TOC is considered as an important factor in the accumulation of hydrophobic organic contaminants in soil^39^. In the present study, the PCB concentrations showed positive and significant correlations with TOC on the windward side (p < 0.05), while there were no such relationships observed on the leeward side (Table S2). For PBDEs, there were also no correlations obtained on both sides of the mountain. This could suggest that trapping of POPs by mountain soil was affected by other factors, rather than just TOC in soil^40^.

**Figure 3:**
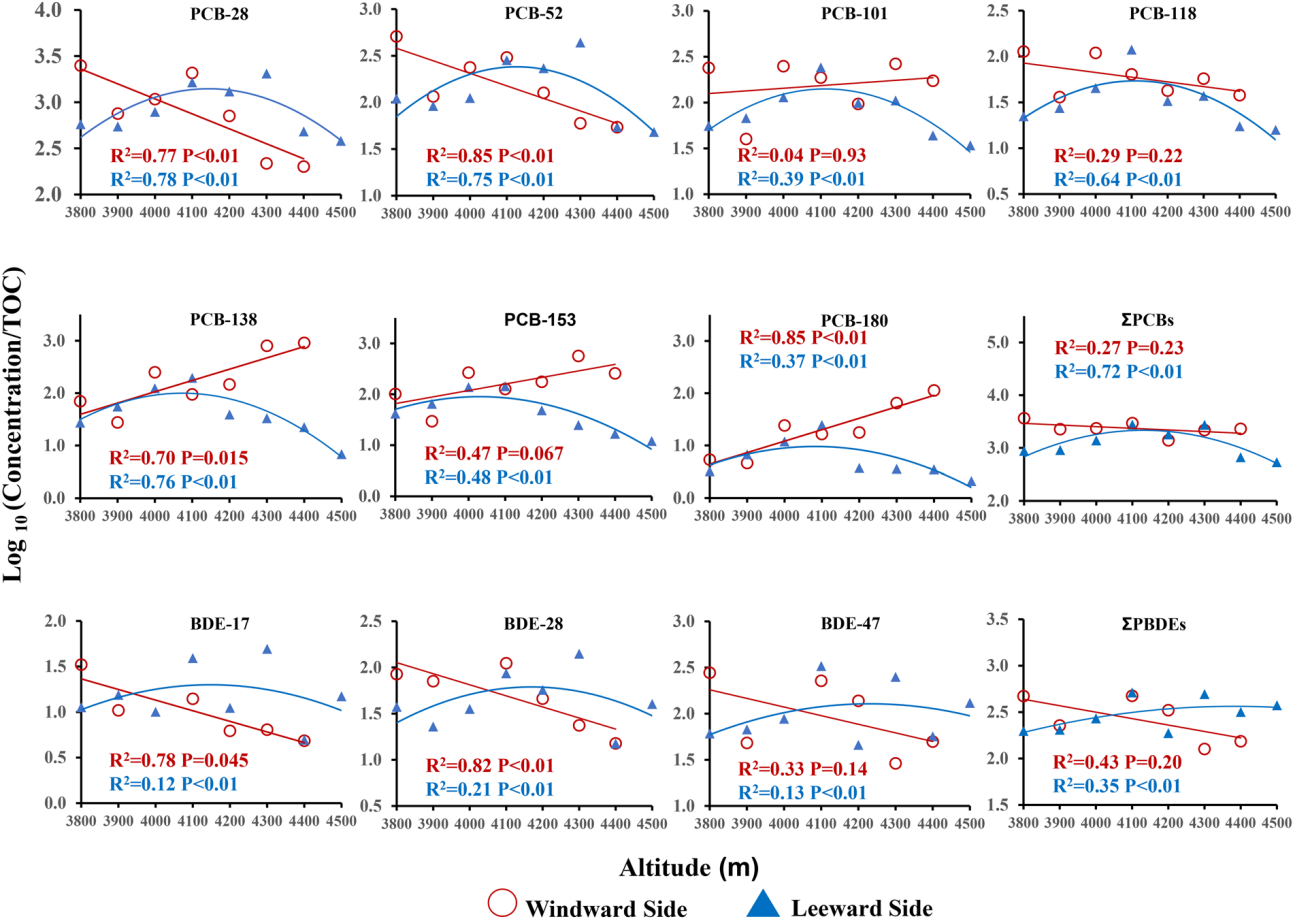
Altitudinal trend of the logarithm TOC-normalized concentrations for selected PCBs and PBDEs along the windward and leeward side of Mt. Sygera.

To examine the relationship between POP concentration and the altitude, the TOC-normalized concentrations were log-transformed, then correlated with the altitudes of the sampling sites. The relationships between POP concentrations and the altitudes showed different trends on the windward and leeward sides. On the windward side of Mt. Sygera, there were positive and significant correlations for heavier congeners (e.g., PCB-138, -153, -180) and altitude (P < 0.05, Fig. 3 and Table S3), which showed an obvious mountain cold-trapping effect^31^. The results were similar to the observations reported in other studies in high mountain regions^12,41,42^. Wania^18^ indicated that heavier POPs were prone to be enriched in high altitudes and it could be well explained by the mountain contamination potential (MCP) model (Fig. S1 and Table S4). Chemicals, of which the log K_OA_ at 25 °C ranged from 8.5 to 11.5 and log K_WA_ ranged from 3.5 to 6, showed higher enrichment at higher altitudes. This phenomenon was mainly caused by the differences in the efficiency of precipitation scavenging at various altitudes for different compounds. Moreover, higher soil–air partition coefficient (K_SA_) have been observed for high molecular weight PCBs (high K_OA_), suggesting that chemicals with high K_OA_ may become enriched in cold climate soil^43^. So, heavier congeners were more prone to be enriched in higher elevation sites with lower temperature. However, the correlation was negative against the altitude for lighter congeners (e.g., PCB-28, -52 and -118, BDE-17, -28 and -47), which was different from the increasing trends of lighter congeners below 4200 m on the leeward side. This difference was expected because the windward side lies windward of the summer Indian monsoon along the valley and is more strongly influenced by the air flow than the leeward side. The lighter congeners were not in the equilibrium controlled by the air-soil system in this region^44^, which coincided with our results that the concentrations of lighter congeners showed no correlation with TOC. The diurnal wind pattern, which was likely to disturb the general upward deposition of POPs, could have stronger influence on the windward side than the leeward side of the mountain^45^.

On the leeward side, the POP concentrations increased with increasing altitude and then the trend was reversed at 4100–4200 m, which fitted a second order polynomial model very well (Fig. 3 and Table S5). This was different from the windward side where all sampling sites are below the timberline, i.e., LS-4300 is at timberline and LS-4400 and LS-4500 are above the timberline. Background soils solely receive input of POPs via atmospheric deposition, which contains two processes in forested area: direct deposition from the atmosphere by precipitation and indirect input by forest filtering^32^. Direct deposition of POPs from the atmosphere mainly resulted from the precipitation. In this region, precipitation increases by 20.9 mm when the altitude increases by 100 m^38^. More precipitation with the increasing altitude would scavenge more POPs from the atmosphere, which would result in an increase in direct deposition into soils. Moreover, indirect input of POPs from the atmosphere into soil was mainly caused by forest filtering. Forest canopies can filter and intercept atmospheric POPs and then transferred them into soil, which was called forest filtering effect (FFE)^46^. FFE is affected by the differential leaf area index (LAI), which can reflect soil concentration differences among forest types. In this study, LS-(3800–4300 m) were dominant in fir forest (forest zone) while LS-(4400–4500 m) in alpine meadow (non-forest zone). Correspondingly, LAI sharply decreased from about 8 (in forest) to 1 (with alpine meadow) along the leeward side^47^. Additionally, it was found that the ratio of POP concentration in forest and non-forest zone was 3.8 ± 2.9 on average (Table S6), which was similar to the results obtained by Meijer *et al*.^27,48^. The capacity of forest filtering POPs from the atmosphere decreased from forest to non-forest region, which resulted in the decrease of indirect deposition into soil. This indicated that the change of forest types resulted in a decrease in concentration, suggesting that forest played a crucial role in the accumulation of POPs.

**Figure 4:**
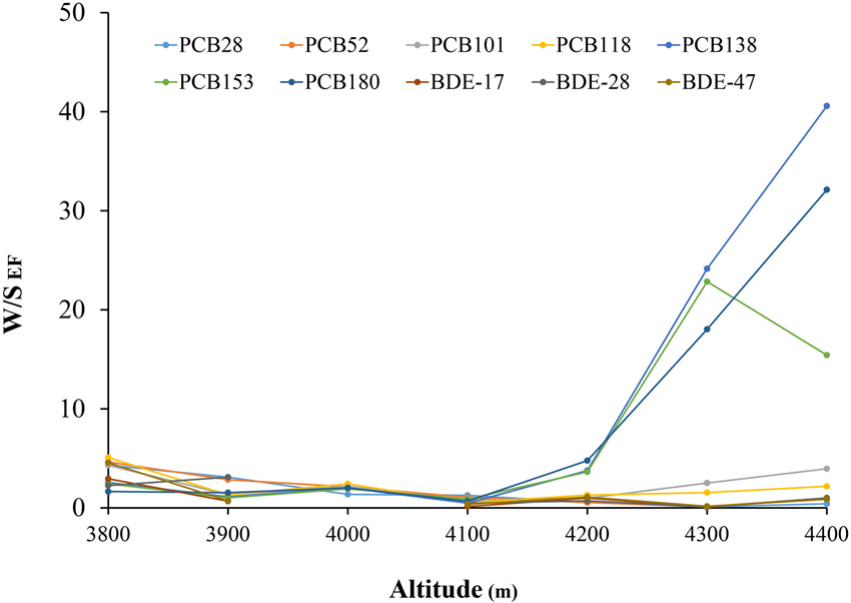
The W/S EFs of different congeners along the altitude in Mt. Sygera.

The ratio of TOC-normalized POP concentrations in soils on the windward and leeward side at the same altitude can be called windward-leeward Enrichment Factor (W/L EF)^35^. These values calculated for all the compounds are reported in Table. S3. W/L EFs were 2.1 (0.77–4.15) for Σ_19_PCBs and 1.5 (0.16–3.04) for Σ_16_PBDEs. Interestingly, W/L EFs decreased with the altitude below 4200 m but then increased especially for heavier congeners (e.g., PCB-138, -153, -180, and -209). It was found that in high elevation sites (4300–4400 m) W/L EFs of heavier congeners (e.g., PCB-138, 153, 180 and 209) sharply increased by one order of magnitude above 4200 m (Fig. 4). That could be attributed to the change of vegetation (Abies and Smithii vs dwarf shrub or alpine meadow) on the leeward side above 4200 m as opposed to the spruce forest (Abies and Juniper) on the windward side. Forest filters the heavier POPs from the atmosphere into the surface soil^25,40,49^, which enforces the accumulation of POPs in soil below 4200 m. Therefore, a shift of W/L EFs further confirmed that forest played an important role in altitudinal accumulation of POPs along different sides of a mountain.

## Methods

### Sampling

A sampling campaign was conducted at 15 sites in Mt. Sygera in August of 2012. Seven soil pits were collected along the windward side and eight soil pits were collected along the leeward sides of the mountain. These sites on the windward side are represented by soil of spruce forest (Abies and Juniper, 3800–4400) while spruce forest (Abies and Smithii, 3800–4200 m), timberline acetone (dwarf shrub, 4300 m) and alpine meadow (4400–4500 m) on the leeward side, respectively. Sampling details and sample characteristics were presented in our previous work^24,25^. All the samples were wrapped by aluminum foil and sealed in the clean plastic bags, then shipped to the laboratory and stored at −20 °C until analysis.

### Sample analysis

Sample extraction, cleanup and chemical analysis followed our previously established method but with some modifications^50^. Detailed procedures are given in the Supplementary Information. The analytical results were obtained based on an isotope dilution method using high-resolution gas chromatography coupled with high-resolution mass spectrometry (HRGC/HRMS). The details were shown in Supplementary Information.

## Electronic supplementary material

Supplementary Information
