# Supplementary material for: Altitudinal dependence of PCBs and PBDEs in soil along the two sides of Mt. Sygera, southeastern Tibetan Plateau
Source: Sci Rep. 2018 Sep 19;8:14037. doi: 10.1038/s41598-018-32093-y (PMC6145894; doi:10.1038/s41598-018-32093-y)
Supplement: Supplementary file 1 — Supplementary Information [file 41598_2018_32093_MOESM1_ESM.docx]

**Supplementary Information**

**Altitudinal dependence of PCBs and PBDEs in soil along the two sides of Mt. Sygera, southeastern Tibetan Plateau**

Wenying Meng ^1, 2^, Pu Wang ^1^, Ruiqiang Yang ^1^, Huizhong Sun ^1, 2^, Julius Matsiko ^1, 2^, Dou Wang ^1, 2^, Zuo Peijie ^1, 2^, Yingming Li ^1^, Qinghua Zhang ^1, 2, 3^ ^[[1]](#footnote-1)^*, Guibin Jiang ^1, 2^

*^1^ State Key Laboratory of Environmental Chemistry and Ecotoxicology, Research Center for Eco- Environmental Sciences, Chinese Academy of Sciences, Beijing 100085, China*

*^2^ University of Chinese Academy of Sciences, Beijing 100049, China*

*^3^ Institute of Environment and Health, Jianghan University, Wuhan 430056, China*

**Figures and Tables**

**Fig S1.** Mountain contamination potential (MCP) value plot of selected compounds.

**Table S1.** The average concentrations of PCBs and PBDEs in soil in Mt. Sygera.

**Table S2.** Statistics of linear regression between concentration (pg g^-1^, dw) and TOC in soil along the windward and leeward sides of Mt. Sygera.

**Table S3.** The regression analysis of Log TOC-normalized concentrations along the altitude on the windward side of Mt. Sygera.

**Table S4.** Physicochemical properties of selected compounds.

**Table S5.** The analysis of Log TOC-normalized concentrations and the altitude on the leeward side of Mt. Sygera.

**Table S6.** The ratio of POP concentrations in soils in spruce forest and alpine meadow on the leeward side of Mt. Sygera.

**Table S7.** W/S EF of every congener at different altitude.

**Materials**

Pesticide grade reagents dichloromethane (DCM) and n-hexane were purchased from Tedia Company Inc. (Fairfield, OH, USA) and nonane was from Sigma (St. Louis，USA). Silica gel 60 (0.063-0.100 mm particle diameter) was from Merck (Darmstadt, Germany), anhydrous sodium sulfate, concentrated sulfuric acid and sodium hydroxide were domestic products at guaranteed grade. Prior to use, silica gel and anhydrous sodium sulfate were baked at 550 ^o^C for 12 h and 660 ^o^C for 6 h respectively. The preparation of acid silica gel and basic silica gel were described elsewhere (Liu et al., 2006). All PCBs and PBDEs surrogate and internal standards were purchased from Wellington Laboratories (Ontario, Canada).

**Sample extraction and cleanup**

The soil samples were freeze-dried, homogenized and sieved through 16-mesh sieve (1.18 mm). 10 grams of dry samples were weighed and extracted with DCM: n-hexane (1:1, v/v) using Accelerating Solvent Extraction (ASE300, Dionex). Prior to extraction, 10 μl 68A-LCS (^13^C_12_-labled PCB congeners) and 10 μl PBDE-LCS (^13^C_12_-BDE-47, -99 and -153) were concurrently spiked to the samples. The conditions of the extraction were as follows: the temperature was 150 ^o^C and the pressure was 1500 psi (10.3 MPa) with 7 min for heating and 8 min in the static state. The extracts were processed solvent exchange with DCM and concentrated to 5 ml, then fractioned on auto GPC (AccuPrep^TM^, J_2_-Scientific) to remove humic substances and sulfur. Subsequently, the collected fractions were solvent exchanged with n-hexane and eluted with 100 ml n-hexane through a multilayer silica column packed from the bottom up with 1 g neutral silica, 4 g basic silica, 1 g neutral silica, 8 g acid silica, 2 g neutral silica and 2 cm anhydrous sodium sulfate. Prior to instrumental analysis, the concentrated eluate was spiked with 68A-IS to determine the recoveries of the ^13^C labeled PCBs and PBDEs.

**Instrumental analysis**

PCBs and PBDEs were analyzed using isotope-dilution method and high-resolution gas chromatography coupled with high-resolution mass spectrometry (HRGC/HRMS). The target compounds included 19 PCB congeners, i.e. 12 dioxin-like PCB congeners (PCB-77, -81, -105, -114, -118, -123, -126, -156, -167, -169, -189), 6 indicator PCB congeners (PCB-28, -52, -101, -138, -153, -180) and PCB-209, and 16 PBDE congeners, i.e. BDE-17, -28, -47, -49, -66, -71, -85, -99, -100, -119, -126, -138, -153, -154 and -183.

The HRGC instrument is an Agilent 6890N (Wilmington，USA) with a CTC PAL autosampler, and the HRMS is AutoSpec Ultima (Waters Micromass，UK) with an electron impact (EI) ion source. Exactly 1μl solution was injected into GC with a 60 m DB-5 fused silica capillary column (J&W, Scientific, 0.25 μm film thickness, 0.25 mm i.d.) for PCBs and 30 m DB-5 column (0.10 μm film thickness, 0.25 mm i.d.) for PBDEs in splitless mode. The HRMS was operated in VSIR mode at R≥10, 000, the electron emission energy was set to 35 eV and the source temperature was 270 ^o^C for PCBs and 280 ^o^C for PBDEs. Helium was the carrier gas with a constant flow of 1.0 ml min^-1^. The temperature conditions of chromatographic analysis were described as follows: PCBs: the initial column temperature was held at 80 ^o^C for 3 min, and then increased to 150 ^o^C at a rate of 15 ^o^Cmin^-1^, after maintaining 150 ^o^C for 2 min, it was increased to 270 ^o^C at 2.5 ^o^Cmin^-1^ and held for 3 min, then heated to 330 ^o^C at 15 ^o^Cmin^-1^ and maintained for 13 min; PBDEs: the initial temperature was 90 ^o^C for 2 min, then increased to 210 ^o^C at 25 ^o^Cmin^-1^ and maintained for 1 min, sequentially, it was elevated to 275 ^o^C at 10 ^o^Cmin^-1^ and held for 10 min; finally increased to 330 ^o^C at 25 ^o^Cmin^-1^ and maintained for 10 min.

**Quality assurance/quality control (QA/QC).**

HRGC/HRMS was employed to analyze the samples combined with isotope-dilution method in the present study. The recoveries of ^13^C-labled surrogated standards in the samples were 65±9% and 56±9%, respectively. The limits of detection (LOD) were defined as three times of signal-to-noise ratio (S/N) and the isotopic ratios between the two main ion pairs were within ±15% of the theoretical value. The LODs were in the range of 0.011-1.023 pg g^-1^ for PCBs and 0.013-0.673 pg g^-1^ for PBDEs. Travel blank (XAD resin) and laboratory blanks were processed in parallel with soil samples to check for potential contamination during the shipment and entire analytical process. The results showed that no contaminants were detected except for several indicator PCBs (e.g., PCB-28 and PCB-52) with a relatively low level in the laboratory blanks (<15% of those in the soil samples). Therefore, the reported concentrations were not corrected for the laboratory blank.


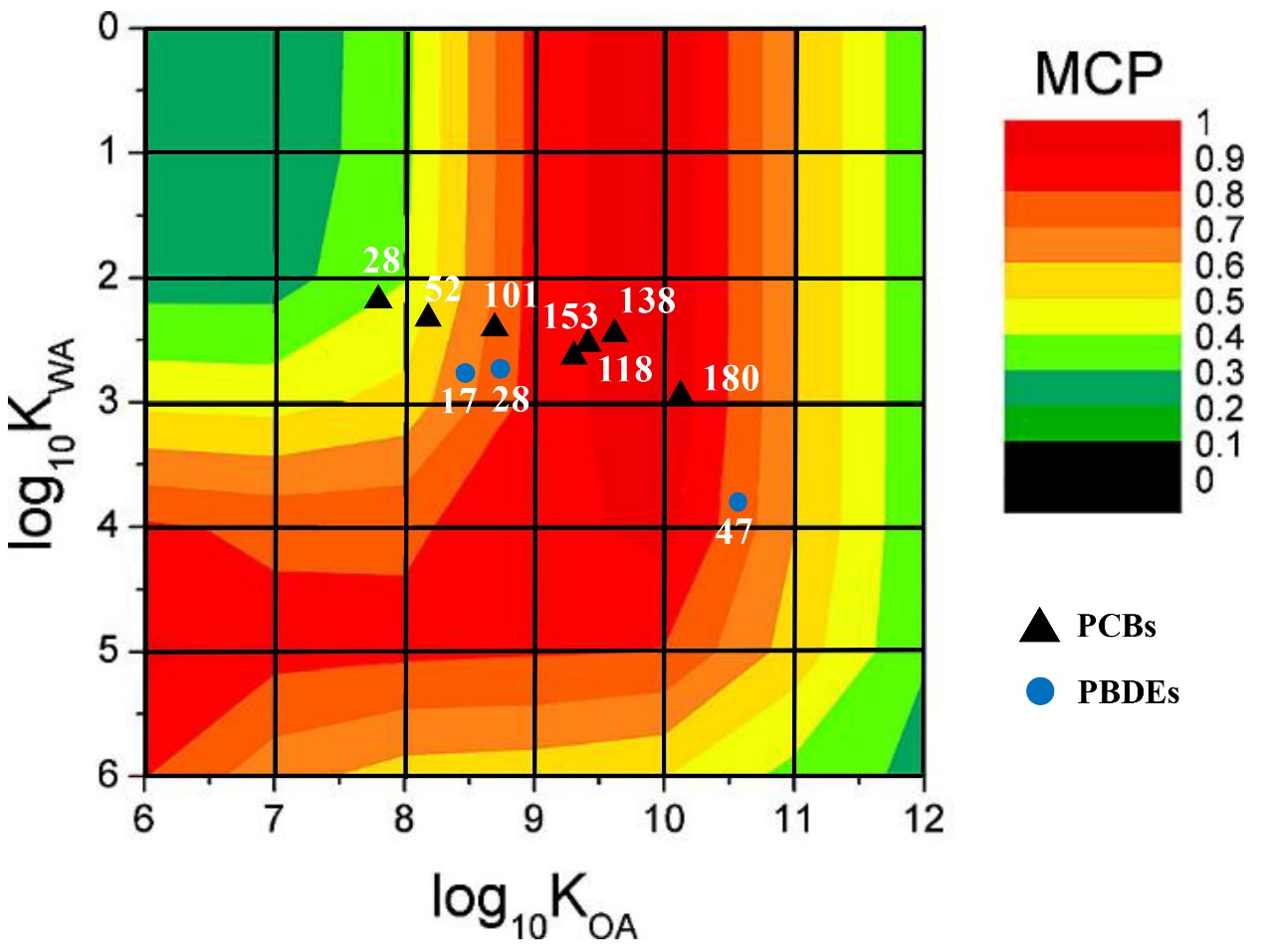


**Fig S1.** Mountain contamination potential (MCP) value plot of selected compounds^1^.

**Table S1.** The average concentrations of PCBs and PBDEs in soil in Mt. Sygera.

| **Compounds** | **Windward side** | | **Leeward side** | | **Total** |
| --- | --- | --- | --- | --- | --- |
|  | **Conc.**  **(pg g^-1^ dw)^a^** | **DF^b^%** | **Conc.**  **(pg g^-1^ dw)^a^** | **DF^b^%** | **Conc.**  **(pg g^-1^ dw)^a^** |
| **PCB77** | 0.87 | 100 | 0.54 | 100 | 0.69 |
| **PCB81** | 0.059 | 29 | 0.011 | 88 | 0.033 |
| **PCB105** | 2.0 | 100 | 1.1 | 100 | 1.5 |
| **PCB114** | 0.17 | **86** | 0.069 | **75** | 0.11 |
| **PCB123** | 0.66 | 100 | 0.31 | 88 | 0.47 |
| **PCB126** | 0.14 | 43 | 0.013 | 25 | 0.070 |
| **PCB156** | 0.32 | 100 | 0.14 | 100 | 0.22 |
| **PCB157** | 0.12 | 100 | 0.057 | 100 | 0.086 |
| **PCB167** | 0.083 | 57 | 0.022 | 25 | 0.050 |
| **PCB169** | 0.81 | 100 | 0.29 | 100 | 0.53 |
| **PCB189** | 0.023 | 29 | 0.32 | 88 | 0.18 |
| **PCB28** | 73 | 100 | 75 | 100 | 73 |
| **PCB52** | 13 | 100 | 13 | 100 | 13 |
| **PCB101** | 15 | 100 | 6.9 | 100 | 11 |
| **PCB118** | 4.8 | 100 | 2.8 | 100 | 3.7 |
| **PCB138** | 33 | 100 | 4.3 | 100 | 18 |
| **PCB153** | 21 | 100 | 4.3 | 100 | 12 |
| **PCB180** | 3.4 | 100 | 0.50 | 100 | 1.9 |
| **PCB209** | 8.8 | 86 | 0.56 | 100 | 4.4 |
| **ΣPCBs** | 177 |  | 110 |  | 141 |
| **BDE-17** | 0.74 | 100 | 1.4 | 100 | 1.1 |
| **BDE-28** | 3.8 | 100 | 4.1 | 100 | 4.0 |
| **BDE-47** | 8.1 | 100 | 9.0 | 100 | 8.6 |
| **BDE-49** | 0.81 | 100 | 0.20 | 63 | 0.58 |
| **BDE-66** | 0.19 | 83 | 0.25 | 88 | 0.22 |
| **BDE-71** | 0.074 | 50 | 0.064 | 25 | 0.068 |
| **BDE-77** | 0.42 | 50 | 0.078 | 63 | 0.23 |
| **BDE-85** | n.d | n.d | n.d | n.d | n.d |
| **BDE-99** | 0.30 | 67 | 0.34 | 63 | 0.32 |
| **BDE-100** | 0.12 | 33 | 0.062 | 25 | 0.086 |
| **BDE-119** | n.d | n.d | n.d | n.d | n.d |
| **BDE-126** | 0.019 | 17 | n.d | n.d | 0.008 |
| **BDE-138** | n.d | n.d | n.d | n.d | n.d |
| **BDE-153** | 0.18 | 33 | n.d | n.d | 0.077 |
| **BDE-154** | 0.17 | 17 | n.d | n.d | 0.072 |
| **BDE-183** | 0.49 | 33 | 0.42 | 50 | 0.45 |
| **ΣPBDEs** | **16** |  | 15 |  | 16 |

a—“pg/g dw” indicateds “pg/g dry weight”;

b—“DF%” indicates “detection frequency (%);

c—“n.d” indicates “non-detectable”

**Table S2.** Statistics of linear regression between concentration (pg g^-1^, dw) and TOC in soil along the windward and leeward sides of Mt. Sygera.

| **Compounds** | **Windward Side** | | | | **Leeward Side** | | | |
| --- | --- | --- | --- | --- | --- | --- | --- | --- |
|  | **N** | **R^2^** | **Slope** | **P** | **N** | **R^2^** | **Slope** | **P** |
| **PCB-77** | 7 | 0.24 | 0.07 | 0.15 | 8 | 0.10 | 0.06 | 0.45 |
| **PCB-105** | 7 | 0.20 | 0.23 | 0.17 | 8 | 0.05 | 0.16 | 0.29 |
| **PCB-156** | 7 | **0.77** | 0.07 | **0.01** | 8 | 0.18 | 0.02 | 0.17 |
| **PCB-157** | 7 | **0.70** | 0.01 | **0.01** | 8 | **0.36** | 0.00 | **0.07** |
| **PCB-169** | 7 | 0.07 | -0.03 | 0.56 | 8 | 0.09 | 0.07 | 0.48 |
| **PCB-28** | 7 | 0.09 | -5.02 | 0.51 | 8 | 0.10 | 9.23 | 0.45 |
| **PCB-52** | 7 | 0.09 | -0.70 | 0.52 | 8 | 0.08 | 1.81 | 0.51 |
| **PCB-101** | 7 | **0.79** | 2.83 | **0.01** | 8 | 0.02 | -0.28 | 0.75 |
| **PCB-118** | 7 | 0.34 | 0.47 | 0.17 | 8 | 0.13 | -0.34 | 0.38 |
| **PCB-138** | 7 | **0.70** | 11.29 | **0.02** | 8 | 0.25 | -0.98 | 0.21 |
| **PCB-153** | 7 | **0.76** | 6.56 | **0.01** | 8 | 0.14 | -0.65 | 0.37 |
| **PCB-180** | 7 | **0.59** | 1.05 | **0.04** | 8 | 0.32 | -0.13 | 0.14 |
| **ΣPCBs** | 7 | **0.71** | 19.54 | **0.02** | 8 | 0.05 | 8.33 | 0.61 |
| **BDE-17** | 6 | 0.01 | -0.01 | 0.88 | 8 | 0.01 | -0.07 | 0.82 |
| **BDE-28** | 6 | 0.00 | 0.03 | 0.95 | 8 | 0.03 | 0.34 | 0.68 |
| **BDE-47** | 6 | 0.00 | -0.12 | 0.90 | 8 | 0.12 | -1.27 | 0.41 |
| **ΣPBDEs** | 6 | 0.03 | 0.57 | 0.73 | 8 | 0.01 | 0.56 | 0.80 |

**Table S3**. The regression analysis of Log TOC-normalized concentrations along the altitude on the windward side of Mt. Sygera.

| Compounds | N | Slope | R^2^ | P |
| --- | --- | --- | --- | --- |
| PCB77 | 7 | -5.2E-4 | 0.43 | 0.067 |
| PCB81 | 2 |  |  |  |
| PCB105 | 7 | -3.3E-4 | 0.07 | 0.47 |
| PCB114 | 6 | -1.1E-3 | 0.86 | P<0.01 |
| PCB123 | 7 | -4.63E-6 | 0.20 | 0.99 |
| PCB126 | 3 | -0.0010 | 0.81 | 0.065 |
| PCB156 | 7 | 5.9E-4 | 0.19 | 0.18 |
| PCB157 | 7 | -2.6E-4 | 0.31 | 0.11 |
| PCB167 | 4 | -1.3E-3 | 0.73 | 0.094 |
| PCB169 | 7 | -1.1E-3 | 0.54 | 0.036 |
| PCB189 | 2 |  |  |  |
| PCB28 | 7 | -1.8E-3 | 0.77 | P<0.01 |
| PCB52 | 7 | -0.0016 | 0.85 | P<0.01 |
| PCB101 | 7 | 3.7E-5 | 0.04 | 0.93 |
| PCB118 | 7 | -5.1E-4 | 0.29 | 0.22 |
| PCB138 | 7 | 1.9E-3 | 0.70 | P<0.01 |
| PCB153 | 7 | 1.0E-3 | 0.47 | 0.067 |
| PCB180 | 7 | 1.9E-3 | 0.85 | 0.003 |
| PCB209 | 6 | 2.4E-3 | 0.44 | 0.090 |
| ΣPCBs | 7 | -3.1E-4 | 0.27 | 0.23 |
| BDE-17 | 6 | -1.2E-3 | 0.78 | 0.045 |
| BDE-28 | 6 | -1.4E-3 | 0.82 | P<0.01 |
| BDE-47 | 6 | -9.6E-4 | 0.33 | 0.14 |
| BDE-49 | 6 | -5.4E-4 | 0.01 | 0.39 |
| BDE-66 | 5 | -8.8E-4 | 0.10 | 0.31 |
| BDE-71 | 3 |  |  |  |
| BDE-77 | 3 | 2.9E-3 | 0.78 | 0.077 |
| BDE-85 | 0 |  |  |  |
| BDE-99 | 4 | -8.4E-4 | 0.82 | 0.062 |
| BDE-100 | 2 |  |  |  |
| BDE-119 | 0 |  |  |  |
| BDE-126 | 1 |  |  |  |
| BDE-138 | 0 |  |  |  |
| BDE-153 | 2 |  |  |  |
| BDE-154 | 0 |  |  |  |
| BDE-183 | 2 |  |  |  |
| ΣPBDEs | 7 | -8.01E-4 | 0.43 | 0.20 |

**Table S4.** Physicochemical properties of selected compounds.

| **Compounds** | **LogK_OA_^a^** | **LogK_OW_^b^** | **LogK_WA_^c^** |
| --- | --- | --- | --- |
| **PCB-28** | 7.85 | 5.66 | 2.19 |
| **PCB-52** | 8.22 | 5.91 | 2.31 |
| **PCB-101** | 8.73 | 6.33 | 2.4 |
| **PCB-118** | 9.36 | 6.69 | 2.67 |
| **PCB-138** | 9.66 | 7.22 | 2.44 |
| **PCB-153** | 9.44 | 6.87 | 2.57 |
| **PCB-180** | 10.16 | 7.16 | 3 |
| **BDE-17** | 8.52 | 5.74 | 2.78 |
| **BDE-28** | 8.71 | 5.98 | 2.73 |
| **BDE-47** | 10.5 | 6.81 | 3.69 |

a- LogK_OA_ for PCBs taken from the work of Li and Wania ^2^; PBDEs taken from

the work of Harner and Shoeib ^3^;

b- LogK_OW_ for PCBs taken from the work of Li and Wania ^2^; PBDEs from the

work of Harner and Shoeib ^3^;

c- LogK_WA_ taken from the formula logK_WA_ = logK_OA_ –log*K*_OW_.

**Table S5**. The analysis of LogTOC-normalized concentrations and the altitude on the leeward side of Mt. Sygera.

| Compounds | N | R^2^ | P | Compounds | N | R^2^ | P |
| --- | --- | --- | --- | --- | --- | --- | --- |
| PCB77 | 8 | 0.10 | P<0.01 | BDE-17 | 8 | 0.16 | P<0.01 |
| PCB81 | 1 |  |  | BDE-28 | 8 | 0.22 | P<0.01 |
| PCB105 | 8 | 0.27 | P<0.01 | BDE-47 | 8 | 0.21 | P<0.01 |
| PCB114 | 6 | 0.02 | 0.56 | BDE-49 | 5 | -0.04 | 0.27 |
| PCB123 | 7 | 0.56 | P<0.01 | BDE-66 | 7 | 0.16 | 0.05 |
| PCB126 | 2 |  |  | BDE-71 | 2 |  |  |
| PCB156 | 8 | 0.34 | 0.12 | BDE-77 | 5 | 0.69 | 0.20 |
| PCB157 | 8 | -0.29 | 0.44 | BDE-85 | 0 |  |  |
| PCB167 | 2 |  |  | BDE-99 | 5 | -0.76 | 0.064 |
| PCB169 | 8 | -0.22 | 0.12 | BDE-100 | 2 |  |  |
| PCB189 | 7 | 0.75 | P<0.01 | BDE-119 | 0 |  |  |
| PCB28 | 8 | 0.51 | P<0.01 | BDE-126 | 0 |  |  |
| PCB52 | 8 | 0.36 | P<0.01 | BDE-138 | 0 |  |  |
| PCB101 | 8 | 0.71 | P<0.01 | BDE-153 | 0 |  |  |
| PCB118 | 8 | 0.50 | P<0.01 | BDE-154 | 0 |  |  |
| PCB138 | 8 | 0.78 | P<0.01 | BDE-183 | 4 |  |  |
| PCB153 | 8 | 0.75 | P<0.01 | ΣPBDEs | 8 | 0.42 | P<0.01 |
| PCB180 | 8 | 0.39 | P<0.01 |  |  |  |  |
| PCB209 | 8 | -0.31 | P<0.01 |  |  |  |  |
| ΣPCBs | 8 | 0.60 | P<0.01 |  |  |  |  |

**Table S6**. The ratio of POP concentrations in soils in spruce forest and alpine meadow on the leeward side of Mt. Sygera. POP concentration in soil in alpine meadow was the average of concentrations at 4400 m and 4500 m.

| Compounds | 3800 m | 3900 m | 4000 m | 4100 m | 4200 m | 4300 m |
| --- | --- | --- | --- | --- | --- | --- |
| PCB28 | 1.50 | 1.01 | 1.53 | 2.48 | 3.67 | 4.80 |
| PCB52 | 2.43 | 1.45 | 1.85 | 3.65 | 5.55 | 8.74 |
| PCB101 | 1.60 | 1.39 | 2.47 | 4.05 | 3.07 | 2.74 |
| PCB118 | 1.53 | 1.35 | 2.33 | 4.74 | 2.42 | 2.31 |
| PCB138 | 1.93 | 2.80 | 6.55 | 8.02 | 2.97 | 2.12 |
| PCB153 | 3.22 | 3.59 | 8.02 | 6.48 | 4.04 | 1.73 |
| PCB180 | 1.26 | 1.87 | 3.48 | 5.57 | 1.56 | 1.26 |
| ΣPCBs | 1.65 | 1.22 | 1.95 | 3.02 | 3.65 | 4.62 |
| BDE-17 | 1.46 | 1.43 | 0.98 | 2.95 | 1.55 | 5.79 |
| BDE-28 | 1.73 | 0.75 | 1.23 | 2.32 | 2.85 | 5.86 |
| BDE-47 | 0.81 | 0.64 | 0.88 | 2.55 | 0.66 | 3.03 |
| ΣPBDEs | 1.23 | 1.02 | 0.89 | 2.38 | 1.10 | 3.68 |

**Table S7**. W/S EF of every congener at different altitude.

| Compounds | 3800 (m) | 3900 (m) | 4000 (m) | 4100 (m) | 4200 (m) | 4300 (m) | 4400 (m) |
| --- | --- | --- | --- | --- | --- | --- | --- |
| PCB77 | 3.7 | 1.9 | 2.2 | 0.76 | 1.6 | 1.0 | 2.4 |
| PCB81 | - | - | - | - | - | - | - |
| PCB105 | 4.4 | 1.4 | 2.6 | 0.48 | 1.8 | 1.4 | 2.4 |
| PCB114 | 6.2 | - | - | 0.76 | 1.5 | 1.5 | 2.0 |
| PCB123 | 4.8 | 1.0 | 2.9 | 0.54 | 1.0 | 2.6 | 3.1 |
| PCB126 | - | - | - | - | - | - | 2.0 |
| PCB156 | 1.6 | 2.1 | 1.7 | 0.42 | 2.4 | 4.5 | 5.4 |
| PCB157 | 2.1 | 2.8 | 2.5 | 0.80 | 2.8 | 3.0 | 4.1 |
| PCB167 | - | - | 1.9 | 1.0 |  |  |  |
| PCB169 | 10.5 | 0.91 | 8.2 | 3.6 | 7.1 | 2.1 | 5.4 |
| PCB189 | 0.20 | 0.31 | - | - | - | - | - |
| PCB28 | 4.3 | 3.1 | 1.4 | 1.3 | 0.54 | 0.11 | 0.42 |
| PCB52 | 4.6 | 2.8 | 2.1 | 1.1 | 0.54 | 0.14 | 1.0 |
| PCB101 | 4.3 | 1.3 | 2.2 | 0.77 | 1.0 | 2.5 | 4.0 |
| PCB118 | 5.1 | 1.3 | 2.4 | 0.54 | 1.3 | 1.5 | 2.2 |
| PCB138 | 2.6 | 1.1 | 2.0 | 0.48 | 3.8 | **24** | **41** |
| PCB153 | 2.4 | 1.0 | 1.9 | 0.88 | 3.6 | **23** | **15** |
| PCB180 | 1.6 | 1.5 | 2.0 | 0.67 | 4.8 | **18** | **32** |
| PCB209 | 2.4 | - | 0.94 | 0.81 | 1.7 | **17** | **112** |
| ΣPCBs | **4.2** | **2.5** | **1.7** | **1.1** | **0.77** | **0.80** | **3.5** |
| BDE-17 | 2.9 | 0.68 | - | 0.14 | 1.0 | 0.076 | 0.98 |
| BDE-28 | 2.3 | 3.1 | - | 0.46 | 0.71 | 0.083 | 1.0 |
| BDE-47 | 4.6 | 0.71 | - | 0.36 | 1.1 | 0.15 | 0.88 |
| BDE-49 | 2.9 | 0.73 | - | - | 13 | - | 3.1 |
| BDE-66 | - | 0.43 | - | - | 4.1 | - | 0.38 |
| BDE-71 | - | - | - | - | - | - | - |
| BDE-77 | - | - | - | - | 31 | - | - |
| BDE-85 | - | - | - | - | - | - | - |
| BDE-99 | 2.3 | 0.55 | - | - | - | - | - |
| BDE-100 | - | - | - | - | - | - | - |
| BDE-119 | - | - | - | - | - | - | - |
| BDE-126 | - | - | - | - | - | - | - |
| BDE-138 | - | - | - | - | - | - | - |
| BDE-153 | - | - | - | - | - | - | - |
| BDE-154 | - | - | - | - | - | - | - |
| BDE-183 | 0.65 |  |  |  |  |  |  |
| ΣPBDEs | **3.0** | **0.91** | **-** | **0.81** | **2.4** | **0.16** | **1.4** |

**References**

1. Wania, F. & Westgate, J. N. On the Mechanism of Mountain Cold-Trapping of Organic Chemicals. *Environ. Sci. Technol.***42**, 9092-9098 (2008).

2. Li N., Wania F., Lei Y. & Daly G.L. *J. Phys. Chem. Ref. Data* **32**, 1545-1590 (2003).

3. Harner T. & Shoeib M. *J. Chem. Eeg. Data* **47**, 228-232 (2002).

1. * Corresponding author. Tel/Fax: +86 10-62849818

   *Email address*: qhzhang@rcees.ac.cn [↑](#footnote-ref-1)
